# Incidence of Cytomegalovirus Primary and Secondary Infection in Adolescent Girls: Results From a Prospective Study

**DOI:** 10.1093/infdis/jiad182

**Published:** 2023-06-21

**Authors:** Robert Paris, Dan Apter, Suresh Boppana, Maria D’Aloia, Nathalie De Schrevel, Jean-Marc Delroisse, Luca Grassano, Adrienne Guignard, Anil A Panackal, Francois Roman, Jing Yu, Elsa M Yunes, Ilse Dieussaert

**Affiliations:** Vaccines, GSK, Rockville, Maryland, US; VL-Medi Clinical Research Center, Helsinki, Finland; Departments of Pediatrics and Microbiology, University of Alabama at Birmingham, Birmingham, US; Vaccines, GSK, Rixensart, Belgium; Vaccines, GSK, Rixensart, Belgium; Vaccines, GSK, Wavre, Belgium; Vaccines, GSK, Siena, Italy; Vaccines, GSK, Wavre, Belgium; Vaccines, GSK, Rockville, Maryland, US; Vaccines, GSK, Wavre, Belgium; Vaccines, GSK, Rockville, Maryland, US; Center for Research on Population Health, National Institute of Public Health, Cuernavaca, Mexico; Vaccines, GSK, Wavre, Belgium

**Keywords:** CMV pp65 qPCR, anti-CMV tegument IgG ELISA, cytomegalovirus, primary infection, secondary infection

## Abstract

Developing a vaccine to prevent congenital cytomegalovirus (CMV) infection and newborn disability requires an understanding of infection incidence. In a prospective cohort study of 363 adolescent girls (NCT01691820), CMV serostatus, primary infection, and secondary infection were determined in blood and urine samples collected at enrollment and every 4 months for 3 years. Baseline CMV seroprevalence was 58%. Primary infection occurred in 14.8% of seronegative girls. Among seropositive girls, 5.9% had ≥4-fold increase in anti-CMV antibody, and 23.9% shed CMV DNA in urine. Our findings provide insights on infection epidemiology and highlight the need for more standardized markers of secondary infection.

## Teaser

We found annual cytomegalovirus reinfection/reactivation rates of 2%–8% and primary infection rates of 5% in adolescent girls in Finland, Mexico, and the United States. Our study highlights the need for standardized markers for reinfection/reactivation.

## BACKGROUND

Congenital cytomegalovirus (cCMV) infection is a major cause of sensorineural hearing loss and neurodevelopmental disabilities [1], with a birth prevalence of 0.2% to 6.2% [2].

In utero transmission of CMV to the fetus can occur after a primary infection in seronegative pregnant women or after a secondary infection in women with preexisting immunity, as a result of reactivation of latent virus or reinfection with a new viral strain [3–5]. Studies have shown that cCMV-related disability occurs both after primary and secondary infection, and in countries with high CMV seroprevalence, the majority of cCMV infections and sequelae occur following maternal secondary CMV infection [6].

Different target populations, including adolescent girls, are being considered for the evaluation of vaccines to prevent cCMV infection following both primary and secondary infection during pregnancy. The aim of the current study was to estimate the incidence of primary and secondary CMV infections in adolescent girls; this information is crucial for the design of vaccine studies in the adolescent population.

## METHODS

### Study Design, Participants, and Procedures

This prospective, multicenter study (ClinicalTrials.gov identifier NCT01691820) was performed between October 2012 and April 2017 in Mexico (1 center), Finland (2 centers), and the United States (US; 2 centers)—3 countries with an estimated CMV seroprevalence of 68% or more in women of childbearing age [7]. The study was conducted in accordance with the Declaration of Helsinki, Good Clinical Practice guidelines, and all applicable regulatory requirements. The protocol (available on https://www.gsk-studyregister.com/en/trial-details/?id=115639), and other study-related documents were approved by the appropriate ethics committees or institutional review boards.

After providing written informed consent/assent, adolescent girls aged 10–17 years were enrolled and followed for 3 years, with site visits every 4 months (Supplementary Figure 1). Inclusion and exclusion criteria are detailed in the Supplementary Material. Blood and urine samples were collected from the participants at each visit (months 0–36) (Supplementary Figure 1). Sociodemographic and behavioral data were collected through a questionnaire at months 0, 12, 24, and 36.

Participants were enrolled regardless of their CMV serostatus (seropositive [S^+^] or seronegative [S^–^]). To determine target enrollment, CMV serostatus was based on results from external laboratories (Supplementary Material). However, for the analyses presented here, baseline serostatus was based on GSK's anti-CMV tegument immunoglobulin G (IgG) enzyme-linked immunosorbent assay (ELISA) in serum (Supplementary Material).

A secondary CMV infection (reinfection or reactivation) in baseline S^+^ participants was defined as either a ≥4-fold increase in serum anti-CMV tegument IgG concentrations between consecutive visits or detection of CMV DNA in urine, in adolescent girls who were CMV DNA-negative in urine at the previous visit. CMV DNA in urine was determined by real-time quantitative polymerase chain reaction (qPCR), based on amplification of the phosphoprotein 65 (pp65) gene (Supplementary Material).

The occurrence of primary CMV infection was defined as the appearance of anti-CMV tegument IgG antibodies in baseline CMV S^–^ adolescent girls (i.e., seroconversion). We also examined sociodemographic or behavioral factors associated with baseline CMV seropositivity, and concordance of the baseline serostatus from testing at the external laboratories with GSK's anti-CMV tegument IgG ELISA.

### Statistical Analysis

The target was to evaluate approximately 200 baseline S^+^ adolescent girls. To achieve this, approximately 240 S^+^ girls had to be enrolled, assuming a dropout rate of 15%–20%. Considering a conservative CMV infection rate of 2% per year, 12 baseline S^+^ participants were expected to have a secondary infection during the 3-year study.

Baseline characteristics were summarized using descriptive statistics. The incidence of secondary infection was evaluated using descriptive statistics on baseline S^+^ participants. The percentage of baseline S^+^ participants with a ≥4-fold increase in serum anti-CMV tegument IgG concentrations between consecutive visits was calculated with 95% confidence intervals (CIs). Although a ≥4-fold increase was used as a proxy for secondary infection, we also examined data on ≥2-fold increase. The percentage of baseline S^+^ participants with the presence of CMV DNA in urine was calculated with 95% CIs, based on detection of CMV DNA load of >0 copies/mL in a sample if the previous sample was negative.

To estimate the incidence of primary CMV infection, we calculated (with 95% CI) the proportion of baseline S^–^ participants with an anti-CMV tegument IgG concentration ≥1.136 EU/mL at any of the follow-up visits.

Concordance of the baseline serostatus results between the external laboratories and GSK's anti-CMV tegument IgG ELISA was evaluated using a McNemar test.

To identify risk factors associated with baseline CMV seropositivity, univariate and multivariate analyses were performed (Supplementary Material).

Statistical analyses were performed using SAS Life Science Analytics Framework (SAS Institute Inc., Cary, North Carolina, US).

## RESULTS

Of the 363 adolescent girls enrolled, 299 completed the study and 64 were withdrawn (Supplementary Figure 2); 210 were CMV S^+^ and 152 were CMV S^–^ at baseline based on GSK's anti-CMV ELISA (serostatus was missing for 1 participant). Hence, baseline CMV seroprevalence was 58% overall and highest in Mexico (75% vs 48% in the US and 38% in Finland) (Supplementary Table 1). The mean age at baseline was similar in the S^+^ and S^–^ groups (Supplementary Table 1).

### Secondary CMV Infections

Anti-CMV tegument IgG results were available for at least 1 postbaseline visit in 204 of 210 baseline S^+^ participants. Twelve of these (5.9% [95% CI, 3.1%–10.0%]) had a ≥4-fold increase in anti-CMV tegument IgG concentration during follow-up (Table 1). A ≥2-fold increase in antibody levels was observed in 36 of 204 participants (17.6% [95% CI, 12.7%–23.6%]).

**Table 1.** Percentage of Baseline Cytomegalovirus (CMV)–Seropositive Participants With Increases in Serum Anti-CMV Tegument Immunoglobulin G Concentrations Between Consecutive Timepoints or With Detection of CMV DNA in Urine Without CMV DNA at the Previous Timepoint, by Visit (Per-Protocol Set)

Timepoint (Study Month)	Serum Anti-CMV Tegument IgG					Urine CMV DNA		
	No.	≥4-Fold Increase		≥2-Fold Increase				
		no.	% (95% CI)	no.	% (95% CI)	No.	no.	% (95% CI)
Month 4	202	2	1.0 (0.1–3.5)	5	2.5 (0.8–5.7)	195	7	3.6 (1.5–7.3)
Month 8	197	1	0.5 (0.0–2.8)	5	2.5 (0.8–5.8)	190	10	5.3 (2.6–9.5)
Month 12	198	3	1.5 (0.3–4.4)	6	3.0 (1.1–6.5)	191	10	5.2 (2.5–9.4)
Month 16	196	0	0.0 (0.0–1.9)	1	0.5 (0.0–2.8)	190	9	4.7 (2.2–8.8)
Month 20	191	2	1.0 (0.1–3.7)	5	2.6 (0.9–6.0)	185	3	1.6 (0.3–4.7)
Month 24	189	2	1.1 (0.1–3.8)	6	3.2 (1.2–6.8)	183	8	4.4 (1.9–8.4)
Month 28	188	1	0.5 (0.0–2.9)	5	2.7 (0.9–6.1)	182	6	3.3 (1.2–7.0)
Month 32	184	1	0.5 (0.0–3.0)	1	0.5 (0.0–3.0)	177	3	1.7 (0.4–4.9)
Month 36	180	0	0.0 (0.0–2.0)	3	1.7 (0.3–4.8)	177	7	4.0 (1.6–8.0)
Up to month 36^a^	204	12	5.9 (3.1–10.0)	36	17.6 (12.7–23.6)	197	47	23.9 (18.1–30.4)

The per-protocol set included all enrolled participants with serology results available and no major protocol deviations (or other reasons) leading to exclusion. No. indicates number of participants with available results at the indicated timepoint; no. indicates number of participants with a ≥4-fold or a ≥2-fold increase in anti-CMV tegument IgG at the indicated timepoint compared to the previous timepoint with available results, or with >0 copies/mL CMV DNA in urine at the indicated timepoint and 0 copies/mL CMV DNA at the previous timepoint with CMV DNA results available.

Abbreviations: CI, confidence interval; CMV, cytomegalovirus; IgG, immunoglobulin G.

^a^Participants with a ≥4-fold or ≥2-fold increase or with >0 copies/mL CMV DNA in urine at the indicated timepoint and 0 copies/mL CMV DNA at the previous timepoint with CMV DNA results available, at either of the timepoints up to (and including) the indicated study month.

Urine CMV PCR results were available for at least 1 postbaseline visit in 197 of 210 S^+^ participants, and CMV shedding was observed at ≥1 timepoint during follow-up in 47 of 197 (23.9% [95% CI, 18.1%–30.4%]) participants (Table 1).

### Primary CMV Infections

Anti-CMV tegument IgG results were available for at least 1 postbaseline visit in 149 of 152 baseline S^–^ participants, with seroconversion observed in 22 of 149 (14.8% [95% CI, 9.5%–21.5%]) participants during follow-up (Table 2).

**Table 2.** Percentage of Baseline Cytomegalovirus (CMV)–Seronegative Participants With Appearance of Serum Anti-CMV Tegument Immunoglobulin G, by Visit (Per-Protocol Set)

Timepoint (Study Month)	no./No.	% (95% CI)
Month 4	5/143	3.5 (1.1–8.0)
Month 8	5/142	3.5 (1.2–8.0)
Month 12	4/142	2.8 (0.8–7.1)
Month 16	6/137	4.4 (1.6–9.3)
Month 20	8/129	6.2 (2.7–11.9)
Month 24	12/134	9.0 (4.7–15.1)
Month 28	8/126	6.3 (2.8–12.1)
Month 32	9/122	7.4 (3.4–13.5)
Month 36	12/113	10.6 (5.6–17.8)
Up to month 36^a^	22/149	14.8 (9.5–21.5)

The per-protocol set included all enrolled participants with serology results available and no major protocol deviations (or other reasons) leading to exclusion. No. indicates number of participants with available results at the indicated timepoint; no. indicates number of participants with anti-CMV tegument IgG ≥1.136 enzyme-linked immunosorbent assay units (EU)/mL at the indicated timepoint.

Abbreviation: CI, confidence interval, CMV, cytomegalovirus, IgG, immunoglobulin G.

^a^Participants with anti-CMV tegument IgG ≥1.136 EU/mL at either of the timepoints up to (and including) the indicated study month.

### Concordance of Baseline Serostatus

Based on GSK's anti-CMV tegument ELISA cutoff of 1.136 EU/mL, 58% of participants were S^+^ at baseline, compared to 60% based on data from external laboratories. Of 211 participants who were S^+^ based on external results, 201 (95%) were also S^+^ based on GSK's ELISA; of 140 participants who were S^–^ based on external results, 137 (98%) were also S^–^ with GSK's ELISA (κ coefficient = 0.92 [95% CI, 0.88–0.96]; Supplementary Table 2).

### Factors Associated With Baseline CMV Seropositivity

The univariate analysis showed that country of residence, ethnicity, school attendance, the number of children (aged <18 years or <3 years) living at home, and the frequency of contact with children aged <3 years not living at home were significant risk factors for baseline CMV seropositivity (Supplementary Tables 3 and 4). In the multivariate analysis, only country of residence remained significantly associated with CMV seropositivity. The odds ratio of being S^+^ at baseline in Mexico vs the US was 3.28 (95% CI, 1.71–6.28; Supplementary Table 5).

## DISCUSSION

This prospective study estimated primary and secondary CMV infections in adolescent girls in Finland, Mexico, and the US. We found that 58% of girls enrolled were S^+^ at baseline, and that during the 3-year follow-up, possible secondary infection occurred in 5.9% based on ≥4-fold antibody increases, and in 23.9% based on CMV DNA shedding in urine, suggesting an annual incidence of CMV secondary infection of approximately 2%–8% in this population. Of the baseline S^–^ girls, 14.8% had seroconverted (5% annual primary CMV infection). These results may provide useful information for future clinical trials of CMV vaccine candidates in terms of calculating sample sizes, determining study duration, and selecting study sites. However, the rates of secondary infection based on serum antibody increase or urinary CMV shedding differed markedly. The discrepant results of the 2 measures to identify secondary CMV infection highlight the need for standardized serological or virological methods to diagnose secondary CMV infection. Lack of tools to distinguish between reinfection or reactivation is another major gap in our understanding of secondary CMV infection. In the absence of a standardized case definition, we considered that the detection of viral DNA in urine (using our specific pp65 PCR assay) and increases in the magnitude of the serological response could serve as possible proxies for potential reinfection or reactivation. A ≥4-fold increase in antibody concentrations was used in a previous study as it is more likely to indicate reinfection/reactivation [8]. Studies that estimated reinfection rates based on the detection of strain-specific antibodies over time showed an annual reinfection rate between 10% and 30% in urban, low-income US and Brazilian S^+^ women [9, 10]. Our estimates based on a ≥4-fold increase in antibody concentrations are lower, possibly because of different sensitivities of the assays used, different intervals between sampling time points, or because of the lower CMV seroprevalence in our study (which may impact reinfection). In contrast, a ≥2-fold increase in antibody levels was seen in 3 times as many participants as a ≥4-fold increase. However, the utility of an antibody titer increase (≥4-fold and ≥2-fold) needs to be evaluated and validated in studies with larger sample sizes.

Our estimate of the incidence of primary infection (5% per year) is in line with previous studies showing annual seroconversion rates of 1%–7% in populations with a CMV seroprevalence ranging between 40% and 80% [11, 12].

The overall baseline CMV seroprevalence in our study (58%) was lower than global, modeled estimates in women of childbearing age (86%), as were the country-specific estimates (Mexico, 75% vs 86%; US, 48% vs 68%; Finland, 38% vs 72%) [7]. This may in part be related to the study participants’ age, with seroprevalence of adolescent girls not yet reaching levels similar to the adult population, or reflect the overall population seroprevalences at the study sites. Additionally, our study was not intended as a representative population survey and the small sample size may have limited the precision of the seroprevalence estimate.

In our multivariate analysis, only the country of residence was an independent risk factor for CMV seropositivity. Although contact with young children at home is a well-established risk factor for CMV infection in pregnant women [13, 14], it was not an independent risk factor in our study. This may reflect different household compositions and roles of adolescent girls compared to adult women. Also, the sample size of our study was too small to identify all risk factors.

A strength of our study is the high concordance between the seroprevalence determined by the GSK anti-CMV tegument IgG ELISA and the commercial assays used in the external laboratories. However, the cutoff for seropositivity may need further validation and optimization. While this was a descriptive study with a relatively small sample size, the targeted enrollment of 200 evaluable CMV S^+^ participants was sufficient to estimate the incidence of secondary infection.

A major limitation of this study is the lack of an established or validated method to reliably estimate secondary CMV infection (reinfection/reactivation). Rises in CMV-specific IgG concentrations in previously S^+^ individuals may not always be detectable after reinfection or reactivation or, if present, may also be due to nonspecific stimulation of the immune system. The estimated secondary infection rates (reinfection/reactivation) were considerably different using the antibody concentration rise versus DNA shedding, highlighting the need to develop and validate standardized measures of secondary infection.

In summary, we found a yearly CMV reinfection/reactivation rate between 2% and 8% and primary infection rate of 5% in adolescent girls in Finland, Mexico, and the US. We found a high concordance between the anti-CMV tegument ELISA and commercial assays to detect CMV-specific antibodies, supporting the use of our assay to determine the CMV serostatus in clinical trials. Our data indicate that primary and secondary CMV infections can be detected with the available tests in adolescent girls, supporting the inclusion of CMV S^+^ and S^–^ adolescent girls in clinical trials to evaluate the efficacy of CMV vaccines. Our findings also demonstrate the need for standardized markers of secondary infection in S^+^ individuals.

## Supplementary Data

Supplementary materials are available at *The Journal of Infectious Diseases* online. Consisting of data provided by the authors to benefit the reader, the posted materials are not copyedited and are the sole responsibility of the authors, so questions or comments should be addressed to the corresponding author.

## Supplementary Material

jiad182_Supplementary_Data

## Notes

***Author contributions.*** S. B.: conduct of the study, collection of data, manuscript writing and review. D. A. and R. P.: conduct of the study, collection and analysis/interpretation of the data, manuscript review. E. M. Y.: design and collection of the data, manuscript review. M. D.: collection of the data, manuscript review. N. D. S.: design of the study, collection and analysis/interpretation of the data, manuscript review. J.-M. D.: design and conduct of the study, collection and analysis/interpretation of the data, manuscript review. J. Y.: conduct of the study, collection and analysis/interpretation of the data, manuscript review. L. G. and A. A. P.: analysis/interpretation of the data, manuscript review. A. G.: design of the study, manuscript review. I. D. and F. R.: design and conduct of the study, collection of the data, manuscript review.

***Acknowledgments.*** The authors are grateful to all the participants. They would like to acknowledge Olivier Gruselle, Thierry Pascal, Dirk van Alewijk, Leontine van der Wel, and Ilona van Haaften for their help with qPCR characterization and clinical testing, as well as Viroclinics-DDL for qPCR testing. They also thank Nathalie Baudson and Yongjia Pu for their contributions to the study and Kirsten Schneider-Ohrum for her critical review of the manuscript. The authors also thank the Akkodis platform for writing support (Natalie Denef) and manuscript coordination, on behalf of GSK.

***Data availability.*** A summary of the results is available on ClinicalTrials.gov (NCT01691820). Anonymized individual participant data and study documents can be requested for further research from www.clinicalstudydatarequest.com.

***Financial support.*** This work was supported by GlaxoSmithKline Biologicals S.A.

***Disclaimer.*** The funding source was involved in all stages of the study conduct and analysis and paid for all costs associated with the development and publishing of this manuscript.
