# Supplementary material for: Incidence of Cytomegalovirus Primary and Secondary Infection in Adolescent Girls: Results From a Prospective Study
Source: J Infect Dis. 2023 Jun 21;228(11):1491–5. doi: 10.1093/infdis/jiad182 (PMC10681855; doi:10.1093/infdis/jiad182)
Supplement: jiad182_Supplementary_Data [file jiad182_supplementary_data.docx]

# Supplementary material

**Inclusion and exclusion criteria**

All participants had to satisfy all the following criteria at study entry:

- A female adolescent between and including 10 and 17 years at the time of enrollment (girls were ineligible for enrollment on their 18th birthday) regardless of pregnancy status and contraception method used or not used.
- Individual for whom the investigator believed that the individual and/or the individual’s parent(s)/legally acceptable representative(s) could and would comply with the requirements of the protocol (e.g., sample collection through self-collection or by a home-visiting nurse, return for follow-up visits).
- Written informed assent obtained from the individual and informed consent obtained from the individual and/or the parent(s)/legally acceptable representative(s) of the individual.
- Individual likely to remain in the area and/or return for required study site visits and complete sample collection visits.

If any of the following exclusion criteria (checked at the time of study entry) applied, the individual was not included in the study:

- Child in care
- Use or planned use of any investigational or non-registered antiviral drug or vaccine during the study period.
- Known medical history of any recurrent clinical herpes episodes requiring episodic or chronic suppressive treatment with oral or parenteral antiviral treatment such as acyclovir, famciclovir, valacyclovir or any other anti-herpes virus antiviral during the year preceding enrollment (no laboratory testing required). Topical antivirals were allowed.
- Individuals with a history of previous vaccination against cytomegalovirus (CMV).
- Chronic administration (defined as more than 14 days in total) of immunosuppressants or other immune-modifying drugs within 6 months prior to visit 1 or planned administration during the study. For corticosteroids, this meant prednisone, 0.5 mg/kg/day, or equivalent. Inhaled and topical steroids were allowed.
- Administration of immunoglobulins and/or any blood products within 3 months prior to visit 1 or planned administration during the study.
- Any confirmed or suspected immunosuppressive or immunodeficient condition including human immunodeficiency virus infection, based on medical history and physical examination (no laboratory testing required).
- Any major congenital defects, serious chronic illness or organ transplantation.

**External laboratories**

Initial categorization as CMV seropositive or seronegative (to inform target enrollment) was done based on serum antibody screening results from either local laboratories (following local practices) or, for US samples, from a designated central laboratory (BARC USA Inc., using DiaSorin Liaison CMV immunoglobulin G and M [IgG and IgM] chemiluminescence immunoassay).

**Anti-cytomegalovirus tegument IgG ELISA**

This indirect solid-phase enzyme-linked immunosorbent assay (ELISA) used two highly purified, autologous fusion proteins, CG1 and CG2, each combining two immunodominant fragments from human CMV tegument (phosphoprotein [pp]150 [UL32]; CG1: amino acids [aa] 495–691 and 862–1048; CG2: aa 695–854) and the delayed-early DNA binding protein (pp52 [UL44], aa 297-433) to capture serum IgG.

The initial assay cut-off (lower limit of quantitation [LLOQ]) of 0.668 ELISA units (EU)/mL was revised to 1.136 EU/mL to account for the inherent assay variability by multiplying 0.668 with the estimated assay variation (1.7).

**Detection of CMV DNA**

The detection of CMV DNA in urine was based on real-time quantitative polymerase chain reaction (qPCR), based on amplification of the pp65 gene. qPCR was performed at DDL Diagnostic Laboratory B.V., Viroclinics DDL, the Netherlands. The amplification of the target sequence was detected using the 5’ nuclease assay based on the TaqMan chemistry. An 82-bp fragment was amplified with the forward primer 5’‑TGTCCCAAAAATATGATAATCAAACC‑3’ and reverse primer 5’‑CAAAATGCTCGTGTGAGGTAAAAG‑3’, and was detected with the probe 5’‑CAAGATCTCGCACATCATGCTGG‑3’.

**Univariate and multivariate analyses**

To identify risk factors associated with baseline CMV seropositivity, univariate and multivariate analyses were performed using CMV serostatus at baseline as the outcome variable. Country, age at month (M) 0, ethnicity, and the various sociodemographic/behavioral factors collected at M0 were used as predictor variables in the univariate analysis. Two-sided t-tests were performed for continuous variables and chi-square tests for categorical variables. Any predictor variable that was associated with CMV infection status with a p-value <0.1 in the univariate model was selected as a covariate for the multivariate analysis. Multivariate analysis was performed using a logistic regression model with both forward and backward stepwise selection. Effects were entered into and removed from the model such that each forward selection step could be followed by one or more backward elimination steps. The stepwise selection process terminated if no further effect could be added to the model or if the current model was identical to a previously visited model. Odds ratios and 95% Wald confidence intervals were estimated from the model.

**Supplementary Table 1. Baseline demographic characteristics of the study participants (enrolled set)**

| **Characteristic** | **CMV S+**  **(N=210)** | **CMV S-**  **(N=152)** |
| --- | --- | --- |
| **Age, years** |  |  |
| Mean ± SD | 13.5 ± 2.1 | 13.4 ± 2.2 |
| Median (min–max) | 13.0 (10–17) | 14.0 (10–17) |
| **Ethnicity, n (%)** |  |  |
| American Hispanic or Latino | 136 (64.8) | 44 (28.9) |
| Not American Hispanic or Latino | 74 (35.2) | 108 (71.1) |
| **Geographic ancestry, n (%)^a^** |  |  |
| Hispanic and mixed race | 137 (65.2) | 50 (32.9) |
| White – Caucasian/European | 64 (30.5) | 93 (61.2) |
| African/African American | 8 (3.8) | 8 (5.3) |
| Southeast Asian | 1 (0.5) | 0 (0.0) |
| White – Arabic/North African | 0 (0.0) | 1 (0.7) |
| **Country, n (%)** |  |  |
| Mexico | 135 (64.3) | 44 (28.9) |
| Finland | 51 (24.3) | 82 (53.9) |
| United States | 24 (11.4) | 26 (17.1) |

^a^Geographic ancestry reflects the participants’ countries of residence: most participants in Mexico were Hispanic and mixed race, while most participants in Finland and the United States were White.

CMV, cytomegalovirus; max, maximum; min, minimum; N, total number of enrolled participants in each group; n (%), number (percentage) of participants in the specified category; S+, seropositive at baseline; S-, seronegative at baseline; SD, standard deviation.

**Supplementary Table 2. Concordance between baseline CMV serostatus determined by external laboratories and by the GSK anti-CMV tegument IgG ELISA using a seropositivity cut-off of 1.136 EU/mL (enrolled set)**

| **Serostatus based on GSK’s anti-CMV tegument IgG ELISA ≥1.136 EU/mL** | **Serostatus based on testing results from external labs** | | |
| --- | --- | --- | --- |
|  | **CMV S+, n (%)** | **CMV S-, n (%)** | **Total, n (%)** |
| **CMV S+, n (%)** | 201 (57.3) | 3 (0.9) | 204 (58.1) |
| **CMV S-, n (%)** | 10 (2.8) | 137 (39.0) | 147 (41.9) |
| **Total, n (%)** | 211 (60.1) | 140 (39.9) | 351 (100) |

Participants with missing serostatus at baseline (in either of the laboratories) were not included.

Kappa coefficient (estimated from the McNemar test) = 0.92, 95% CL: 0.88–0.96.

CMV, cytomegalovirus; CL, confidence limit; ELISA, enzyme-linked immunosorbent assay; EU, enzyme-linked immunosorbent assay units; IgG, immunoglobulin G; n (%), number (percentage) of participants in the specified category; S+, seropositive at baseline based on the specified test; S-, seronegative at baseline based on the specified test.

**Supplementary Table 3. Summary of baseline socio-demographic and behavioral factors possibly associated with baseline CMV seropositivity (per-protocol set)**

| **Variable at baseline (M0)** | **CMV S+**  **(N=208)** | | **CMV S-**  **(N=152)** | | **Total**  **(N=360)** | | **P-value^a^** |
| --- | --- | --- | --- | --- | --- | --- | --- |
|  | **n** | **%** | **n** | **%** | **n** | **%** |  |
| **Country** |  |  |  |  |  |  |  |
| Finland | 51 | 24.5 | 82 | 53.9 | 133 | 36.9 | <0.0001 |
| Mexico | 133 | 63.9 | 44 | 28.9 | 177 | 49.2 | - |
| United States | 24 | 11.5 | 26 | 17.1 | 50 | 13.9 | - |
| **Age in categories^b^** |  |  |  |  |  |  |  |
| <12 years old | 39 | 18.8 | 39 | 25.7 | 78 | 21.7 | 0.1161 |
| ≥12 years old | 169 | 81.3 | 113 | 74.3 | 282 | 78.3 | - |
| **Ethnicity^c^** |  |  |  |  |  |  |  |
| American Hispanic or Latino | 134 | 64.4 | 44 | 28.9 | 178 | 49.4 | <0.0001 |
| Not American Hispanic or Latino | 74 | 35.6 | 108 | 71.1 | 182 | 50.6 | - |
| **Do you go to school** |  |  |  |  |  |  |  |
| No | 11 | 5.3 | 2 | 1.3 | 13 | 3.6 | 0.0451 |
| Yes | 196 | 94.7 | 150 | 98.7 | 346 | 96.4 | - |
| Missing | 1 | - | 0 | 0.0 | 1 | - | - |
| **Number of adults at home** |  |  |  |  |  |  |  |
| ≤1 | 20 | 9.6 | 22 | 14.5 | 42 | 11.7 | 0.1904 |
| 2 | 123 | 59.1 | 93 | 61.2 | 216 | 60.0 | - |
| ≥3 | 65 | 31.3 | 37 | 24.3 | 102 | 28.3 | - |
| **Number of children <18 years at home** |  |  |  |  |  |  |  |
| 0 | 38 | 18.3 | 46 | 30.5 | 84 | 23.4 | 0.0030 |
| 1 | 78 | 37.5 | 62 | 41.1 | 140 | 39.0 | - |
| Other | 92 | 44.2 | 43 | 28.5 | 135 | 37.6 | - |
| Missing | 0 | - | 1 | - | 1 | - | - |
| **Number of children <3 years at home** |  |  |  |  |  |  |  |
| 0 | 175 | 84.5 | 140 | 92.1 | 315 | 87.7 | 0.0308 |
| Other | 32 | 15.5 | 12 | 7.9 | 44 | 12.3 | - |
| Missing | 1 | - | 0 | - | 1 | - | - |
| **Do children <3 years go to day care** |  |  |  |  |  |  |  |
| No | 26 | 83.9 | 10 | 83.3 | 36 | 83.7 | 0.9658 |
| Yes | 5 | 16.1 | 2 | 16.7 | 7 | 16.3 | - |
| Missing | 177 | - | 140 | - | 317 | - | - |
| **Type of things you do with children <3 years living at home** | | | | | | | |
| Contact^d^ | 31 | 100 | 12 | 100 | 43 | 100 | - |
| Missing | 177 | - | 140 | - | 317 | - | - |
| **Frequency of contact with children <3 years not living at home** | | | | | | | |
| A few times per month | 42 | 20.4 | 40 | 26.5 | 82 | 23.0 | <0.0001 |
| A few times per week or every day | 115 | 55.8 | 43 | 28.5 | 158 | 44.3 | - |
| Never or a few times per year | 49 | 23.8 | 68 | 45.0 | 117 | 32.8 | - |
| Missing | 2 | - | 1 | - | 3 | - | - |
| **Type of things you do with children <3 years not living at home** | | | | | | | |
| Contact^e^ | 171 | 83.8 | 122 | 81.3 | 293 | 82.8 | 0.5398 |
| Non-contact | 33 | 16.2 | 28 | 18.7 | 61 | 17.2 | - |
| Missing | 4 | - | 2 | - | 6 | - | - |

^a^Based on two-sided t-test for continuous variables and chi-square test for categorical variables.

^b^Age was included in the multivariate analysis (despite having a p-value of 0.12) but did not remain significantly associated with CMV infection.

^c^Ethnicity was not included in the multivariate analysis since it strongly correlated with country.

^d^Contact may correspond to activities such as changing diapers (n=15), feeding the child (n=20), watching the child (n=32) or playing with the child (n=35).

^e^Contact may correspond to activities such as changing diapers (n=49), feeding the child (n=72), watching the child (n=141) or playing with the child (n=269).

CMV, cytomegalovirus; M0, study month 0; N, number of participants in the per-protocol set; n/%, number/percentage of participants in the specified category, with % calculated among participants with available results; S+, seropositive at baseline; S-, seronegative at baseline.

**Supplementary Table 4. Summary of baseline social and behavioral factors possibly associated with baseline CMV seropositivity in Finland and Mexico (per-protocol set)**

| **Variable at baseline (M0)** | **CMV S+**  **(N=184)** | | **CMV S-**  **(N=126)** | | **Total**  **(N=310)** | | **P-value^a^** |
| --- | --- | --- | --- | --- | --- | --- | --- |
|  | **n** | **%** | **n** | **%** | **n** | **%** |  |
| **Country^b^** |  |  |  |  |  |  |  |
| Finland | 51 | 27.7 | 82 | 65.1 | 133 | 42.9 | <0.0001 |
| Mexico | 133 | 72.3 | 44 | 34.9 | 177 | 57.1 | - |
| **Age in categories** |  |  |  |  |  |  |  |
| <12 years old | 32 | 17.4 | 32 | 25.4 | 64 | 20.6 | 0.0872 |
| ≥12 years old | 152 | 82.6 | 94 | 74.6 | 246 | 79.4 | - |
| **Ethnicity^c^** |  |  |  |  |  |  |  |
| American Hispanic or Latino | 133 | 72.3 | 44 | 34.9 | 177 | 57.1 | <0.0001 |
| Not American Hispanic or Latino | 51 | 27.7 | 82 | 65.1 | 133 | 42.9 | - |
| **Do you have children** |  |  |  |  |  |  |  |
| No | 178 | 98.9 | 125 | 100 | 303 | 99.3 | 0.2370 |
| Yes | 2 | 1.1 | 0 | 0.0 | 2 | 0.7 | - |
| Missing | 4 | - | 1 | - | 5 | - | - |
| **Number of children** |  |  |  |  |  |  |  |
| 1 | 2 | 100 | 0 | 0.0 | 2 | 100 | - |
| Missing | 182 | - | 126 | - | 308 | - | - |
| **Age of first child** |  |  |  |  |  |  |  |
| <1 year old | 2 | 100 | 0 | 0.0 | 2 | 100 | - |
| Missing | 182 | - | 126 | - | 308 | - | - |
| **Age of youngest child** |  |  |  |  |  |  |  |
| Missing | 184 | - | 126 | - | 310 | - | - |
| **Have you shared a drink/toothbrush/lip balm or kiss someone in the last 12 months?** | | | | | | | |
| No | 28 | 15.4 | 12 | 10.0 | 40 | 13.2 | 0.1767 |
| Yes | 154 | 84.6 | 108 | 90.0 | 262 | 86.8 | - |
| Missing | 2 | - | 6 | - | 8 | - | - |
| **How many different persons have you kissed in the last 12 months?** | | | | | | | |
| Between 1 and 5 | 68 | 98.6 | 45 | 93.8 | 113 | 96.6 | 0.1598 |
| >5 | 1 | 1.4 | 3 | 6.3 | 4 | 3.4 | - |
| Missing | 115 | - | 78 | - | 193 | - | - |
| **Have you ever had sexual intercourse?** | | | | | | | |
| No | 70 | 78.7 | 51 | 77.3 | 121 | 78.1 | 0.8375 |
| Yes | 19 | 21.3 | 15 | 22.7 | 34 | 21.9 | - |
| Missing | 95 | - | 60 | - | 155 | - | - |
| **How old were you when you had sexual intercourse for the first time?** | | | | | | | |
| <14 years old | 0 | 0.0 | 1 | 6.7 | 1 | 2.9 | 0.2533 |
| ≥14 years old | 19 | 100 | 14 | 93.3 | 33 | 97.1 | - |
| Missing | 165 | - | 111 | - | 276 | - | - |
| **How many different persons have you had sexual intercourse with?** | | | | | | | |
| 1 | 11 | 57.9 | 9 | 60.0 | 20 | 58.8 | 0.9014 |
| >1 | 8 | 42.1 | 6 | 40.0 | 14 | 41.2 | - |
| Missing | 165 | - | 111 | - | 276 | - | - |
| **How many times did you have sexual intercourse in the last 12 months?** | | | | | | | |
| >10 times | 9 | 47.4 | 6 | 40.0 | 15 | 44.1 | 0.6675 |
| Other | 10 | 52.6 | 9 | 60.0 | 19 | 55.9 | - |
| Missing | 165 | - | 111 | - | 276 | - | - |
| **How often did your partner use a condom?** | | | | | | | |
| All the time (100%) | 4 | 22.2 | 5 | 33.3 | 9 | 27.3 | 0.7678 |
| Most of the time (51%–99%) | 6 | 33.3 | 4 | 26.7 | 10 | 30.3 | - |
| Other (0%–50%) | 8 | 44.4 | 6 | 40.0 | 14 | 42.4 | - |
| Missing | 166 | - | 111 | - | 277 | - | - |

^a^Based on two-sided t-test for continuous variables and chi-square test for categorical variables.

^b^The social and behavioral data presented in this table were not collected in the United States due to their personal nature.

^c^Ethnicity was not included in the multivariate analysis since it strongly correlated with country.

CMV, cytomegalovirus; M0, study month 0; N, number of participants in the per-protocol set; n/%, number/percentage of participants in the specified category, with % calculated among participants with available results; S+, seropositive at baseline; S-, seronegative at baseline.

**Supplementary Table 5. Odds ratios of baseline socio-demographic and behavioral factors in multivariate analyses to predict baseline CMV seropositivity (per-protocol set)**

| **Variable** | **Odds ratio (95% Wald CI)** |
| --- | --- |
| **Country** |  |
| Finland vs United States | 0.674 (0.350–1.298) |
| Mexico vs United States | 3.275 (1.708–6.280) |

CI, confidence interval, estimated from stepwise logistic regression model; CMV, cytomegalovirus.


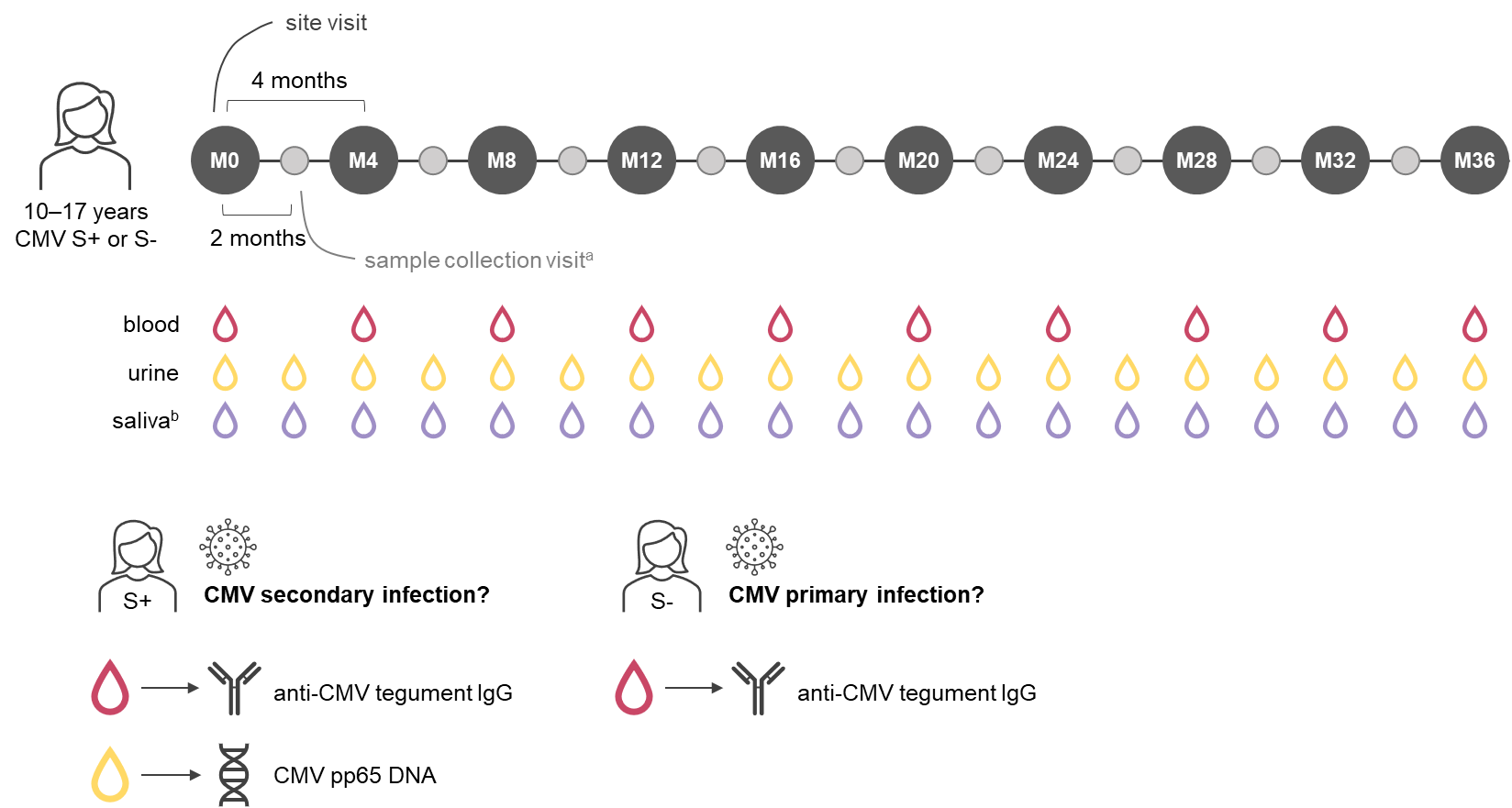


**Supplementary Figure 1. Study design**

^a^The primary and secondary objectives were based on data from site visits; data from sample collection visits were used for various exploratory analyses (not included in the present article).

^b^Data from saliva samples from adolescent girls were used for exploratory analyses (not included in the present article).

CMV, cytomegalovirus; IgG, immunoglobulin G; M, study month; pp65, phosphoprotein 65; S+, seropositive at baseline (i.e., anti-CMV tegument IgG ≥1.136 EU/mL); S-, seronegative at baseline (i.e., anti-CMV tegument IgG <1.136 EU/mL).


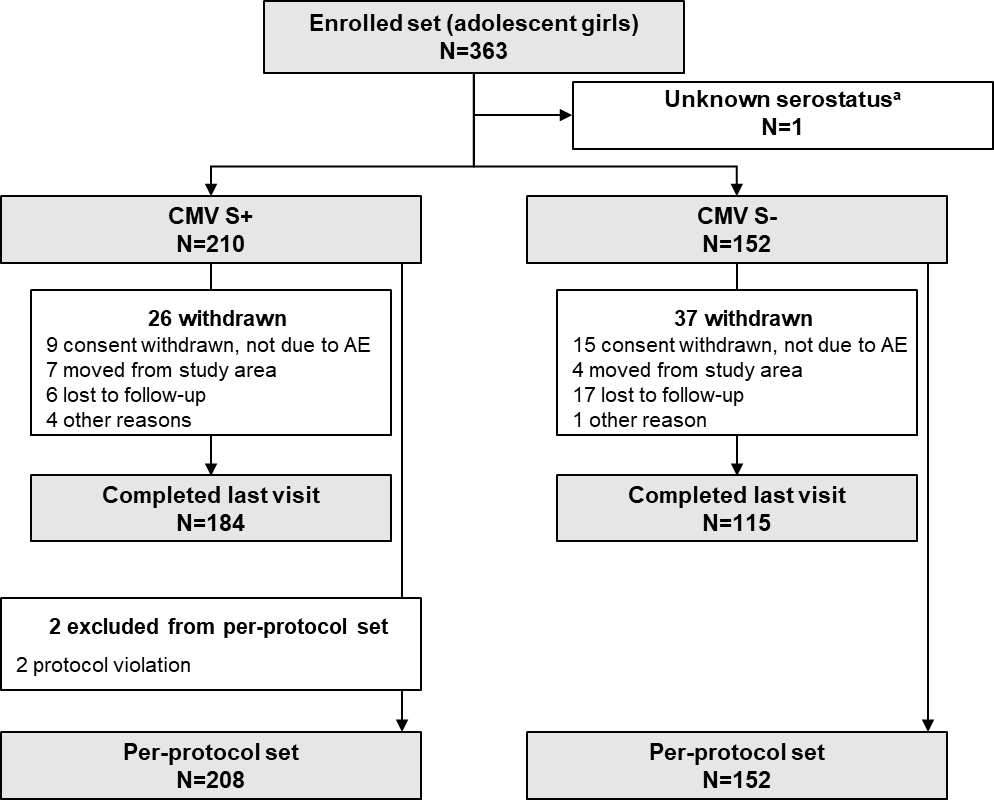


**Supplementary Figure 2. Participant flow diagram**

Baseline serostatus was based on GSK’s anti-CMV tegument IgG ELISA.

^a^The participant with unknown serostatus at baseline did not complete the study and was excluded from the per-protocol set due to missing essential serological data.

AE, adverse event; CMV, cytomegalovirus; N, number of participants; S+, seropositive at baseline (i.e., anti-CMV tegument immunoglobulin G [IgG] ≥1.136 EU/mL); S-, seronegative at baseline (i.e., anti-CMV tegument IgG <1.136 EU/mL).
